# Multifactorial modeling of nematode–soil interactions associated with citrus decline and implications for sustainable orchard management in southern Iran

**DOI:** 10.1038/s41598-026-57077-1

**Published:** 2026-06-11

**Authors:** Hadi Karimipour Fard, Mohammad Mehdi Faghihi, Masoud Latifian, Mohammad Reza Chakeralhosseini, Seyedeh Najmeh Banihashemian

**Affiliations:** 1 Plant Protection Research Department, Kohgiluyeh and Boyerahmad Agricultural and Natural Resources Research and Education Center, AREEO, Yasouj, Iran; 2 Plant Protection Research Department, Fars Agricultural and Natural Resources Research and Education Center, AREEO, Shiraz, Iran; 3 Temperate Fruits Research Center, Horticultural Sciences Research Institute, Agricultural Research Education and Extension Organization (AREEO), Karaj, Iran; 4 Department of Soil and Water Research, Kohgiluyeh and Boyerahmad Agricultural and Natural Resources Research and Education Center, AREEO, Yasouj, Iran; 5 Citrus and Subtropical Fruits Research Center, Horticultural Science Research Institute, Agricultural Research Education and Extension Organization (AREEO), Ramsar, Iran

**Keywords:** Statistical modeling, Soil physicochemical properties, Integrated management, Biotic–abiotic interactions, Ecology, Ecology, Environmental sciences, Plant sciences

## Abstract

Citrus decline, one of the major challenges in the orchards of Fars and Kohgiluyeh–Boyerahmad provinces, is a multifactorial disorder exacerbated by complex interactions between biotic and abiotic factors. This study aimed to model the interactions between the citrus nematode (*Tylenchulus semipenetrans*) and the physicochemical properties of the soil, and to evaluate their combined roles in the severity of tree decline. A total of 80 soil and root samples were collected between 2020 and 2022 using standard nematological methods. The data were analyzed using advanced statistical approaches, including ordinal logistic regression (OLR), generalized additive models (GAMs), and partial least squares–discriminant analysis (PLS-DA). The results revealed a significant relationship between nematode population density and decline severity, with soil texture, salinity, saturation percentage, pH, and lime content (Total Neutralizing Value, TNV) acting as moderating factors. The highest nematode densities were observed in light-textured soils with an electrical conductivity ranging from 2.5 to 3.5 dS m⁻¹. Moreover, a significant synergistic interaction was identified among alkaline pH, high TNV, and nematode population density in inducing micronutrient deficiencies. These findings emphasize the necessity of adopting integrated management strategies, including the use of tolerant rootstocks, nematode population regulation, and soil quality improvement, to mitigate citrus decline losses.

## Introduction

Citrus is among the most important horticultural crops both globally and in Iran, playing a crucial role in the agricultural economy and food security^1^. According to the latest data from the Food and Agriculture Organization (FAO), Iran produces over 632,000 tons of citrus annually, ranking fourth among the world’s citrus producers^2^. Among Iranian provinces, the southern regions—particularly Fars and Kohgiluyeh and Boyer-Ahmad—are considered among the important provinces for citrus production. The total cultivated area in Fars Province covers 61,701 ha, yielding approximately 1,368,015 tons per year. In contrast, Kohgiluyeh and Boyer-Ahmad province has 5,584 ha under citrus cultivation, with a production of around 45,665 tons annually^3^. However, in recent years, citrus decline has emerged as one of the most serious challenges threatening orchard sustainability in these regions^4^.

This complex disorder, characterized by the gradual weakening or sudden death of trees, not only reduces the quantity and quality of fruit yield but also significantly shortens the economic lifespan of orchards. Field observations indicate that in some citrus orchards of Fars province, the damage caused by this phenomenon reaches 40–60%, posing a direct threat to growers’ livelihoods and potentially impacting regional food security in the long term^5^.

Global research has shown that citrus decline is a multifactorial syndrome resulting from the complex interplay between biotic and abiotic agents^6,7^. Among the biotic factors, the citrus root nematode (*Tylenchulus semipenetrans*) is of particular importance. This nematode disrupts root function, hampers water and nutrient uptake, and predisposes trees to secondary infections^8,9^. Studies have reported that infestation by this nematode alone may reduce yield by 30–50%^10^.

Among the abiotic drivers, soil physicochemical characteristics such as salinity, pH, texture, Total Neutralizing Value (TNV), and nutrient availability play a decisive role in either aggravating or mitigating citrus decline. Heavy-textured soils with poor drainage create favorable conditions for nematode proliferation and pathogen activity. Conversely, soils with low organic matter content may exacerbate these conditions by reducing soil structure stability and microbial diversity, which are crucial for suppressing soil-borne pathogens. Whereas high salinity and alkaline soils interfere with nutrient uptake, thereby reducing tree vigor and resistance to biotic stress^11^. Soil organic matter can play a mitigating role in such conditions by improving nutrient availability and buffering against pH extremes^12^.

Several studies worldwide have focused on modeling the interactions between nematodes and soil properties^13^. For example, nematode population density has been linked to soil nitrogen and potassium contents^14^, and significant correlations between soil characteristics and the population of the citrus nematode have been reported^15^. Likewise, soil texture and drainage have been shown to directly influence nematode abundance^16^. Soil organic matter, which is closely linked to texture, salinity, and pH, also plays a critical role in modulating the dynamics of soil-borne pathogens by affecting soil structure, microbial activity, and nutrient availability^17^.

Recent advances in nematode ecology suggest that changes in soil physicochemical conditions can directly affect nematode migration, feeding behavior, and reproduction. Moreover, physiological evidence indicates that soil-related stresses may weaken the host plant’s resistance against nematode infection^18,19^. These findings collectively emphasize the necessity of studying soil and nematode factors simultaneously to better understand the mechanisms underlying citrus decline.

However, to date, no comprehensive study in Iran has modeled the concurrent interactions between nematodes and soil, and their combined effects on citrus decline under field conditions. Most domestic studies have examined either nematode populations or soil parameters in isolation, whereas scientific evidence emphasizes the importance of evaluating these interactions holistically to understand the multifactorial nature of citrus decline.

Integrated management strategies, such as the use of resistant rootstocks, biological control agents and environmentally friendly practices, are recognized as effective approaches for controlling plant-parasitic nematodes^9,20^. These strategies demonstrate the practical relevance of understanding nematode–soil interactions for sustainable orchard management. The significance and necessity of this research are important from several aspects. First, citrus decline causes significant economic losses for citrus growers. Estimates indicate that this phenomenon incurs substantial direct and indirect annual losses for citrus orchards^21^. Second, a clearer understanding of the interrelationships among contributing factors will facilitate the development of targeted management strategies. Third, modeling these interactions provides a predictive framework for assessing the progression of decline and adopting proactive management interventions^22^.

Accordingly, this study aims to elucidate the relationships between citrus nematode populations and soil physicochemical characteristics and to evaluate how their interactions influence the severity of citrus decline in orchards of Fars and Kohgiluyeh and Boyer-Ahmad provinces. Specifically, the study investigates the association between nematode density and key soil parameters, including texture, salinity, pH, organic matter, and nutrient content, as well as the moderating effects of soil properties on nematode-induced decline and symptom intensification.

## Materials and methods

### Comprehensive study design and sampling strategy

This study was conducted as a cross-sectional analytical study between 2020 and 2022 in citrus orchards of Fars and Kohgiluyeh and Boyer-Ahmad provinces. The two study provinces in southern Iran exhibit distinct climatic characteristics due to their complex topography. Fars Province features a diverse climate, ranging from cold mountainous types in the north to warm and semi-arid conditions in the central and southern regions, with mean annual temperatures between 15 and 22 °C and precipitation of 200–350 mm. In contrast, Kohgiluyeh and Boyer-Ahmad is predominantly mountainous with a cold Mediterranean to semi-humid climate, receiving higher annual precipitation (400–700 mm) and experiencing lower mean temperatures (14–18 °C). These climatic differences influence soil formation processes and physicochemical properties, thereby contributing to the regional variations in citrus decline severity observed in this study. Study sites were selected using purposive sampling based on multiple criteria, including the prevalence of decline symptoms, ecological variability, soil heterogeneity, and geographic distribution. Field sampling for this study was conducted during two distinct peak periods of nematode activity over a two-year period from 2020 to 2022. Specifically, sampling was carried out in the early spring (April–May) and autumn (October–November) of each year, coinciding with optimal soil moisture and temperature conditions for nematode and root activities. These seasonal time points were selected to capture maximum nematode population densities and to ensure a representative assessment of soil physicochemical properties under typical climatic conditions in the two provinces.

From each orchard, ten symptomatic trees exhibiting clear decline symptoms and five completely healthy trees were selected as controls. Soil samples were collected from two depths (0–30 cm and 30–60 cm) within the tree canopy area at an appropriate distance from the trunk. Simultaneously, root samples were collected from active feeder roots. At each depth, five independent point samples were combined to form a homogeneous composite sample. In total, 40 soil and 40 root samples were collected and subjected to comprehensive laboratory analyses. Soil and root samples were collected from commercial citrus orchards across multiple counties in Fars and Kohgiluyeh–Boyerahmad provinces. Among these, the highest nematode population densities were recorded in Jahrom county (Fars Province), followed by Basht and Gachsaran counties (Kohgiluyeh–Boyerahmad province).

All root and soil samples were collected from privately owned, fenced commercial citrus orchards with the permission of the orchard owners. The local Agricultural Jihad Organization of Fars and Kohgiluyeh–Boyerahmad provinces also approved the sampling activities. The orchard owners were fully informed about the purpose of the study and voluntarily granted access to their orchards. In return, after laboratory analyses, we provided each orchard owner with a detailed report on the nematode infestation status and soil health indicators of their orchard, which was particularly important given the high infestation levels observed in many of these orchards. The study complied with the national guidelines of Iran for non‑experimental field sampling on cultivated plants. Since Citrus is a well‑known commercially cultivated genus and no endangered or protected species were involved, no additional permits from protected area authorities were required.

### Advanced laboratory methods and physicochemical assessments

Soil nematodes were extracted using the tray method, following the standard Whitehead and Hemming protocol^23^. For accurate enumeration of second-stage juveniles (J2s), 250 mL of each soil sample was processed for 48 h under controlled conditions. Adult females were counted by staining 1 g of root samples with 1% acid fuchsin and examining them under an advanced compound light microscope^24^.

Key soil physicochemical properties were assessed using established standard methods. Soil texture was determined by the hydrometer method, pH, and electrical conductivity (EC) in a 1:2 soil-to-water extract, organic carbon by the Walkley-Black method, total nitrogen using the Kjeldahl method, available phosphorus by Olsen’s method, available potassium by flame photometry, and calcium carbonate equivalent by titration. All measurements were conducted in triplicate to ensure accuracy^25^.

### Advanced statistical modeling and hypothesis testing

#### Hypothesis 1: relationship between nematode density and decline severity

In this study, we employed a sequential hypothesis-testing framework to systematically disentangle the complex interactions underlying citrus decline. For Hypothesis 1, we specifically aimed to first establish the fundamental bivariate relationship between nematode density and decline severity using ordinal logistic regression. However, as outlined in subsequent hypotheses (Hypotheses 2–5), we explicitly incorporated all soil physicochemical indicators—including texture, salinity, saturation percentage, pH, and TNV—through advanced modeling approaches (GAMs, interaction models, and PLS-DA). This stepwise approach allowed us to quantify direct nematode effects, then examine how soil properties modify this relationship, and finally identify latent components governing infection status. While integrating all variables into a unified model is valuable^12^, our sequential framework provides clearer interpretation of the distinct roles and interactions among biotic and abiotic factors.

An ordinal logistic regression model was used to test this hypothesis. The model was formulated as:

$${\mathrm{P}}\left( {{\text{Y }} \le {\text{ j}}} \right) \, = {\mathrm{exp}}\left( {{\uptheta }\_{\text{j }} - {\upbeta }^{{\mathrm{T}}} {\mathrm{X}}} \right) \, / \, \left[ {{1 } + {\text{ exp}}\left( {{\uptheta }\_{\text{j }} - {\upbeta }^{{\mathrm{T}}} {\mathrm{X}}} \right)} \right]$$

where: Y is the ordinal response variable with three levels of citrus decline severity coded as: 1: Healthy (no visible decline symptoms). 2: Early Decline (mild symptoms including reduced vigor, slight leaf chlorosis, and minimal dieback), 3: Gradual Decline (severe symptoms including extensive dieback, severe chlorosis, reduced fruit yield, and tree weakening), θ_j are the threshold parameters, β is the vector of regression coefficients, X is the matrix of predictors, including the logarithm of juvenile and adult nematode densities.

Model fit was evaluated using the Brant test and McFadden’s pseudo-R². Model sensitivity and robustness were assessed via bootstrap-based cross-validation^26^.

#### Hypothesis 2: moderating role of soil texture

Soil texture was analyzed individually as a moderating variable because it functions as a fundamental physical property that governs nematode movement, reproduction, and host accessibility. Unlike chemical properties that directly affect nematode physiology, texture physically influences pore size distribution, water retention, and aeration—factors critical for nematode mobility and survival. Therefore, we specifically tested its moderating effect using isometric log-ratio (ILR) transformation within an interaction model to examine how texture modifies the nematode–decline relationship, rather than treating it as merely another additive predictor. This approach aligns with our objective to dissect interaction mechanisms rather than build a single predictive model.

Two nematode-related variables were selected as predictors based on established biological criteria: (1) log-transformed second-stage juvenile (J2) density per 250 mL of soil, representing the mobile infective stage that invades roots and reflects current soil infestation pressure; and (2) log-transformed adult female density per gram of root, representing the established reproducing population within root tissue and indicating chronic infection intensity. Both variables were included because they capture complementary aspects of nematode infestation dynamics.

The moderating effect of soil texture was analyzed using a compositional data framework with isometric log-ratio (ILR) transformation of soil texture components^27^. The ILR transformation was defined as:

$$z\_i = {\surd} ({\mathrm{i}}/({\mathrm{i}} + 1)){\mathrm{ln}}(\prod \{ k = 1\}^{i} \, x\_k/x \{ i + 1\})$$

The ILR-transformed soil texture components represent specific compositional balances between particle size fractions. ILR1 primarily reflects the contrast between sand content and the geometric mean of silt plus clay (i.e., sand versus fine particles), with higher ILR1 values indicating sandier textures. ILR2 represents the balance between silt and clay within the fine particle fraction, with higher values indicating silt-dominated rather than clay-dominated fine materials.

A regression model incorporating interaction terms was fitted as follows:

$${\upeta } = {\upbeta}_{{0}} \, + \, \sum {\upbeta }\_{\mathrm{iX}}\_{\text{i }} + \, \sum {\upgamma }\_{\mathrm{jZ}}\_{\text{j }} + \, \sum {\updelta }\_{\mathrm{ijX}}\_{\mathrm{iZ}}\_{\text{j }} + {\upvarepsilon }$$

where: X_i represents nematode-related variables (J2 and adult densities), Z_j denotes ILR-transformed soil texture components, and δ_ij are the interaction coefficients.

#### Hypothesis 3: nonlinear effects of soil salinity and saturation percentage

GAMs were selected because preliminary data exploration indicated that relationships between soil salinity, saturation percentage, and nematode density were unlikely to follow simple linear patterns. GAMs offer flexibility by allowing the data to determine the functional form through smooth functions, rather than imposing a priori linear assumptions. This approach is particularly appropriate when ecological thresholds or optimum ranges are expected. Management or prediction strategies based on linear assumptions about these relationships would therefore be unreliable.

To investigate nonlinear relationships, generalized additive models (GAMs) with smoothing functions were employed.

$${\mathrm{g}}\left( {\upmu } \right) \, = {\upbeta}_{{0}} \, + \, \sum {\mathrm{f}}\_{\mathrm{j}}\left( {{\mathrm{x}}\_{\mathrm{j}}} \right) \, + {\upvarepsilon }$$

where f_j are smooth functions estimated using optimal regression spline bases:

$${\mathrm{f}}\_{\mathrm{j}}\left( {\mathrm{x}} \right) \, = \, \sum {\upbeta }\_{\text{k b}}\_{\mathrm{k}}\left( {\mathrm{x}} \right)$$

with b_k(x) representing spline basis functions. The degree of smoothing was optimized using the generalized cross-validation (GCV) criterion^28^.

#### Hypothesis 4: synergistic effects of soil pH and TNV on micronutrient deficiency

To test this hypothesis, a binary logistic regression model with interaction terms was employed:

$${\mathrm{logit}}\left( {{\mathrm{P}}\left( {{\mathrm{Y}} = {1}} \right)} \right) \, = {\upbeta}_{0} \, + {\upbeta}_{1} {\mathrm{x}}_{1} \, + {\upbeta}_{2} {\mathrm{x}}_{2} \, + {\upbeta}_{3} {\mathrm{x}}_{3} \, + {\upbeta}_{4} {\mathrm{x}}_{1} {\mathrm{x}}_{3} \, + {\upvarepsilon }$$

where: x₁ represents soil pH, x₂ denotes TNV (%), x₃ corresponds to nematode density, and the interaction terms (x₁x₃ and x₂x₃) capture synergistic effects.

Binary logistic regression was selected because the outcome variable—presence or absence of micronutrient deficiency symptoms—is binary, and the inclusion of interaction terms allows formal testing of whether soil properties and nematodes act synergistically rather than additively^29^.

#### Hypothesis 5: identification of latent soil components and nematode infestation status

Partial least squares discriminant analysis (PLS-DA) was necessary for dimensionality reduction to identify underlying soil components governing infection status—a task that ordinal regression cannot perform.

While a unified ordinal logit model including all predictors could predict decline severity, it would not adequately test the specific nonlinear, synergistic, and latent structure hypotheses that were central to our research objectives. Each statistical method was selected to match the specific hypothesis and data structure.

To extract latent components, partial least squares discriminant analysis (PLS-DA) was employed. The PLS-DA model is defined as follows^30^:

$${\mathrm{X}}\, = \,{\mathrm{TP}}^{{\mathrm{T}}} + {\text{ E}}$$

$${\mathrm{Y}}\, = \,{\mathrm{UQ}}^{{\mathrm{T}}} + {\text{ F}}$$

where: T and U are the score matrices, P and Q are the loading matrices, and E and F are the residual matrices.

PLS-DA was selected over alternative methods for specific reasons: unlike principal component analysis (PCA), which maximizes variance explanation without considering group structure, PLS-DA explicitly maximizes separation between predefined groups (economically infested vs. non-infested orchards). Unlike linear discriminant analysis (LDA), which requires more variables than samples and assumes normal distributions, PLS-DA handles correlated predictors and smaller sample sizes effectively. Unlike logistic regression, which treats variables as independent predictors, PLS-DA identifies latent components that capture shared variance among soil properties, revealing underlying dimensional structure. This makes PLS-DA particularly suitable for identifying which soil property combinations collectively define infestation risk.

### Supplementary statistical analyses and sensitivity assessments

Multivariate analysis of variance (MANOVA) was employed to compare the mean values of soil physicochemical properties (texture, pH, EC, TNV, organic matter) between samples collected from healthy trees and those from trees exhibiting decline symptoms. Discriminant analysis was used to classify soil and root samples into infestation categories (low vs. high nematode density) based on their physicochemical characteristics. Path analysis was applied to investigate causal relationships among soil properties, nematode density, and decline severity. Bayesian methods were used to estimate parameters under uncertainty and provide robust confidence intervals for the key predictors identified in the ordinal logit model (Hypothesis 1). Sensitivity analyses were conducted using Monte Carlo simulations to evaluate the robustness of results. All statistical analyses were performed in R version 4.1.0 using specialized packages. Type I error was controlled via Bonferroni correction, and statistical power was assessed using Monte Carlo simulations. Power analysis was additionally conducted with G*Power, considering medium effect sizes and a significance level of 0.05 ^31^.

## Results

### Preliminary field observations

Initial field surveys revealed spatial variation in the prevalence of the citrus root nematode (*T. semipenetrans*) across southern Iran. The highest nematode densities were observed in Jahrom County, Fars province, with an average of 3,767 s-stage juveniles (J2) per 250 mL of soil and approximately 67 adult females per gram of root. In Kohgiluyeh and Boyer-Ahmad province, approximately 80% of samples were infested, although in many orchards nematode densities remained below the economic threshold.

Notably, orchards with heavier soil textures, alkaline pH, and higher TNV exhibited more pronounced decline symptoms. To further elucidate the relationships among nematode density, soil physicochemical properties, and tree decline severity, comprehensive multivariate statistical analyses and advanced modeling were subsequently performed, as detailed below.

### Relationship between nematode density and citrus decline severity

Testing of Hypothesis 1 revealed a significant positive association between nematode density and the severity of citrus decline. The ordinal logistic regression model exhibited good fit (− 2 Log Likelihood = 457.32, *P* < 0.001) and a McFadden’s pseudo R² of 0.67, indicating strong explanatory power.

Results indicated that a one-unit increase in the logarithm of second-stage juvenile (J2) density increased the odds of a tree progressing to the gradual decline stage by 3.45-fold. Similarly, a one-unit increase in the logarithm of adult female density raised the odds of gradual decline by 2.89-fold. Both nematode life stages thus showed significant positive associations with decline severity, with J2 density demonstrating a slightly stronger effect. Detailed regression coefficients and corresponding significance levels are provided in Table 1.

**Table 1:** Ordinal logistic regression results for predicting decline severity based on nematode density.

Predictor	Coefficient	Std. error	Wald	*P*-value	Odds ratio
Threshold 1	4.123	0.678	37.890	0.001	-
Threshold 2	2.345	0.567	17.123	0.001	-
Log (J2)	1.237	0.234	27.901	0.001	3.45
Log (adult)	1.061	0.198	28.734	0.001	2.89

**Fig. 1:**
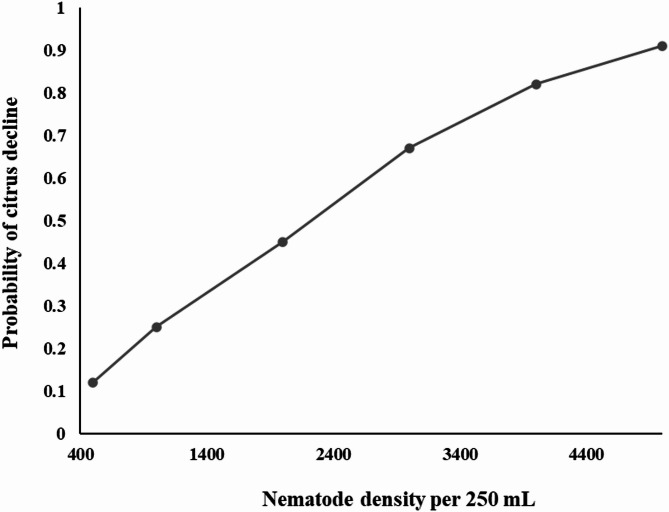
Relationship between second-stage juveniles (J2s) density and the probability of gradual citrus decline.

Figure 1 illustrates the relationship between second-stage juveniles (J2s) density and the probability of gradual citrus decline. As observed, the probability of gradual decline rises exponentially when J2s density exceeds 2,000 individuals per 250 mL of soil.

### Moderating effect of soil texture on the nematode–decline relationship

Hypothesis 2 testing indicated that soil texture significantly moderated the relationship between nematode density and decline severity. Multivariate analysis of variance (MANOVA) revealed a significant interaction effect between nematode density and ILR-transformed soil texture components (Pillai’s Trace = 0.456, *P* < 0.001).

ILR transformation results showed that the sand-to-clay ratio had the strongest moderating effect on the nematode–decline relationship. The interaction coefficient between log-transformed J2s density and the first ILR component was 0.789 (*P* = 0.003), indicating that the nematode effect on tree decline was amplified in lighter-textured soils (Table 2).

**Table 2:** Interaction effects of soil texture and nematode density on decline severity.

Interaction	Coefficient	Std. error	t-value	*P*-value
J2s × ILR1	0.789	0.234	3.372	0.003
J2s × ILR2	0.456	0.189	2.413	0.024
Adult × ILR1	0.678	0.215	3.153	0.005
Adult × ILR2	0.345	0.176	1.960	0.063

ILR1 emerged as the primary soil composition balance shaping nematode impact across both J2 and adult stages, as evidenced by its significant interactions with both life stages (J2s × ILR1: coefficient = 0.789, *P* = 0.003; Adult × ILR1: coefficient = 0.678, *P* = 0.005). This indicates that the sand-to-fine particle balance most strongly moderates the nematode–decline relationship. Furthermore, J2 interactions were consistently significant across both ILR components, while the adult × ILR2 interaction was not significant (*P* = 0.063), suggesting that early-stage nematodes (J2s) are more sensitive to soil composition contrasts than adult females. This differential sensitivity likely reflects the greater dependence of mobile infective juveniles on soil pore structure for movement and host finding, whereas established adult females within roots are partially buffered from physical soil conditions.

**Fig. 2:**
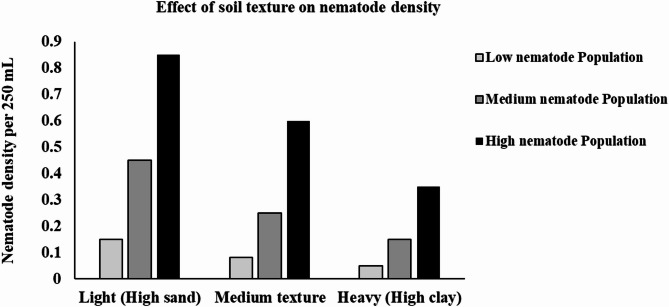
Interaction effect of second-stage juveniles (J2s) density and soil texture on the probability of gradual decline.

Figure 2 illustrates the interaction effect of nematode density and soil texture on the probability of gradual decline. In lighter-textured soils (higher sand content), even moderate nematode densities markedly increased the likelihood of gradual decline.

### Nonlinear effects of salinity and saturation percentage

GAM modeling revealed significant nonlinear relationships between soil salinity, saturation percentage, and nematode density. The final model demonstrated good fit, with an effective degree of freedom (EDF) of 7.89, F = 23.45, *P* < 0.001, and a generalized cross-validation (GCV) score of 456.7 (Table 3).

**Table 3:** Results of the generalized additive model for nonlinear relationships.

Variable	EDF	F	*P*-value	Explained variance
Salinity	3.45	18.90	0.001	34.5%
Saturation (%)	2.78	22.34	0.001	41.2%
Interaction	1.66	8.90	0.012	12.3%

Analysis of the smoothing functions revealed an inverse parabolic relationship between salinity and J2 density, with peak densities observed at 2.5–3.5 dS/m. Furthermore, the relationship between saturation percentage and J2 density was significantly negative, with nematode densities declining markedly when saturation exceeded 45%. From a soil management perspective, these findings suggest that moisture control (reflected by saturation percentage) should be considered the primary factor regulating nematode populations, with salinity management as a secondary but still important consideration.

**Fig. 3:**
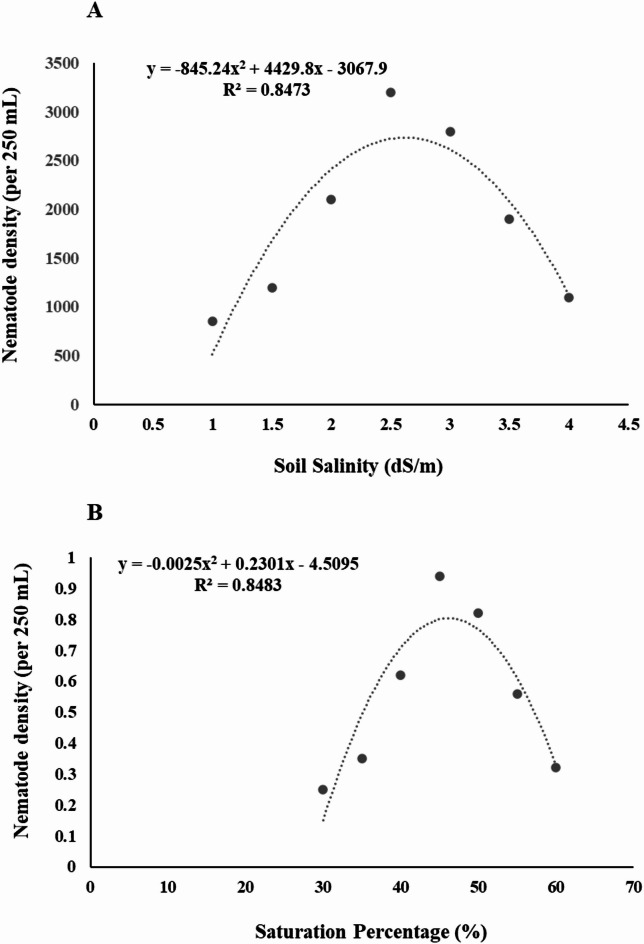
Nonlinear relationships between soil salinity (A), saturation percentage (B), and second-stage juveniles (J2s) density.

Figure 3 illustrates the nonlinear relationships between soil salinity, saturation percentage, and J2 density. The smoothing curves clearly depict the nonlinear patterns in these relationships.

### Synergistic effects of pH and lime on micronutrient deficiency in the presence of nematodes

Binary logistic regression analysis revealed significant synergistic effects of soil pH, TNV, and nematode density on the occurrence of micronutrient deficiency symptoms. The final model demonstrated high discriminative ability (AUC = 0.894; 95% CI: 0.856–0.928).

A significant interaction between pH and nematode density was detected. At pH values above 7.5 and J2 densities exceeding 2,000 per 250 mL of soil, the odds of zinc deficiency symptoms increased 4.56-fold. In soils with TNV greater than 40%, the odds increased 3.23-fold (Table 4).

**Table 4:** Interaction effects of soil properties and nematode density on micronutrient deficiency.

Predictor	Coefficient	Std. error	Odds ratio	*P*-value
pH	1.234	0.345	3.45	0.004
TNV (%)	0.987	0.289	2.68	0.012
Nematode density	1.456	0.321	4.29	0.001
pH × nematode	1.567	0.412	4.78	0.001
TNV × nematode	1.178	0.367	3.25	0.008

A significant interaction between pH and nematode density was detected. At pH values above 7.5 and J2 densities exceeding 2,000 per 250 mL of soil, the odds of zinc deficiency symptoms increased 4.56-fold. In soils with TNV greater than 40%, the odds increased 3.23-fold. These results indicate that farm areas with higher TNV are substantially more likely to experience micronutrient deficiency, and that soil chemistry and nematode density jointly amplify this risk. Collectively, these findings imply that effective deficiency management should combine soil amendment strategies (such as pH adjustment and targeted fertilization) with nematode control measures, rather than addressing either factor in isolation.

**Fig. 4:**
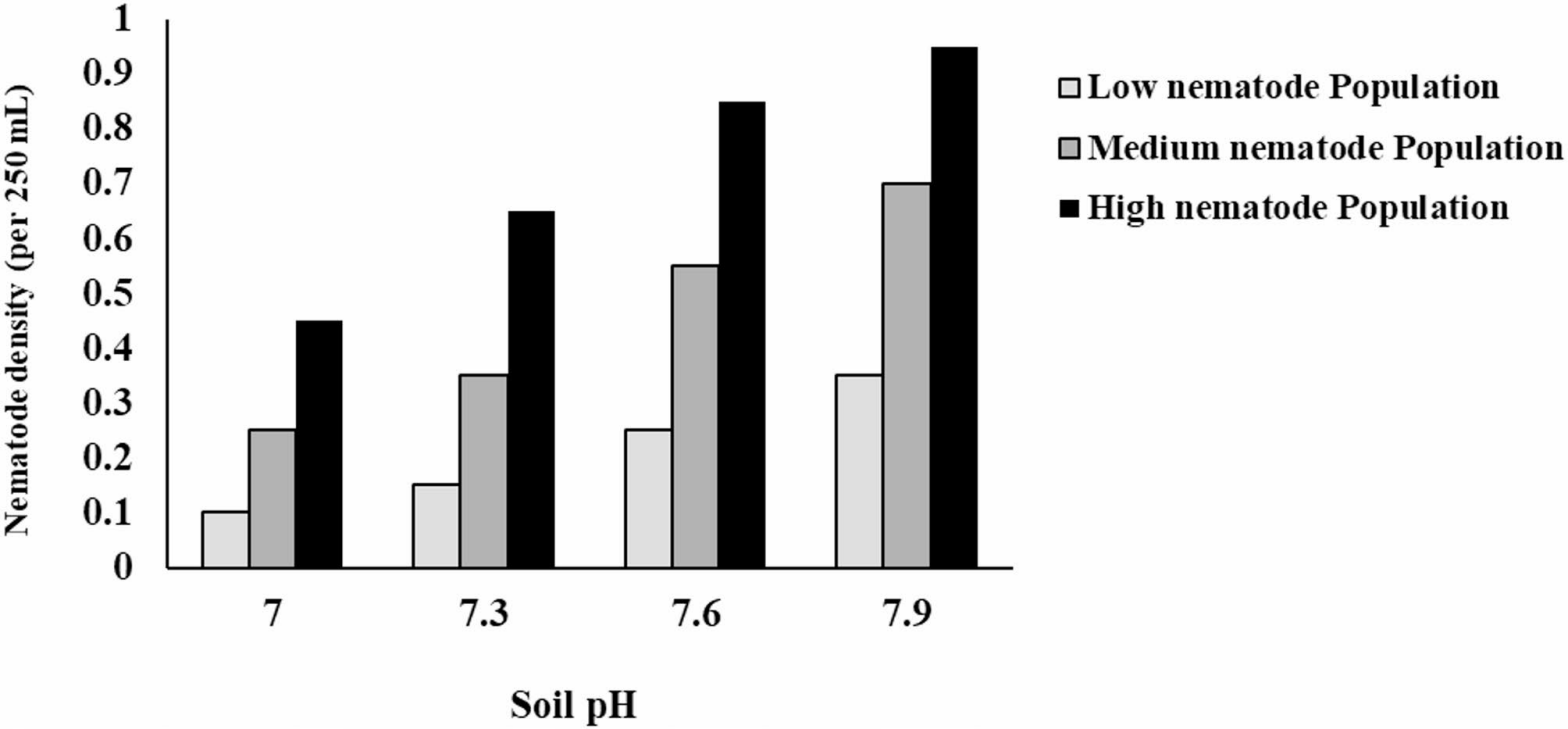
Interaction effect of soil pH and second-stage juveniles (J2s) density on the probability of micronutrient deficiency.

Figure 4 illustrates the interaction between soil pH and nematode density on the probability of micronutrient deficiency symptoms. As shown, in alkaline soils, even moderate nematode densities markedly increased the likelihood of deficiency symptoms.

### Identification of latent soil components influencing economic infection levels

Partial least squares discriminant analysis (PLS-DA) identified two principal latent components that together explained 72.3% of the total variance in the dataset. Component 1, primarily associated with physical soil properties such as texture and saturation percentage, accounted for 48.7% of the variance, while Component 2, linked to chemical soil properties including pH, salinity, and nutrient content, explained 23.6% of the variance.

Examination of the loading matrices revealed that in Component 1, sand (loading = 0.896), saturation (loading = − 0.783), and clay (loading = 0.678) had the strongest contributions. In Component 2, pH (0.812), salinity (0.765), and potassium (− 0.654) were the most influential variables, highlighting their critical roles in determining the economic level of nematode infection (Table 5).

**Table 5:** Loading matrix of principal components from PLS-DA analysis.

Soil variable	Component 1	Component 2	Component 3
Sand (%)	0.896	0.123	0.056
Silt (%)	0.789	0.234	0.098
Clay (%)	0.812	0.189	0.076
Saturation (%)	−0.783	0.145	0.067
Salinity	0.234	0.765	0.123
pH	0.189	0.812	0.145
Lime (%)	0.215	0.789	0.167
Potassium	0.178	−0.654	0.198
Organic C	0.167	0.567	0.789

Examination of the loading matrices revealed that in Component 1, sand (loading = 0.896), saturation (loading = − 0.783), and clay (loading = 0.678) had the strongest contributions, indicating that soil texture and lower saturation are strongly associated with infestation status. In Component 2, pH (0.812), salinity (0.765), and potassium (− 0.654) were the most influential variables, highlighting their critical roles in determining the economic level of nematode infection.

These findings carry important management implications. First, because physical soil properties (texture and saturation) dominated the primary latent component, chemical amendments alone may not be sufficient to reduce infestation risk if structural conditions remain favorable for nematode movement and survival. Second, the strong association between lower saturation and infestation suggests that physical soil management—particularly improving drainage and reducing water retention in light-textured soils—may matter more than single chemical adjustments. Integrated management should therefore prioritize physical soil modification alongside chemical and biological control measures.

Projection of the samples in the principal component space revealed that economically infested samples were predominantly located in the quadrant characterized by high Component 1 values and moderate Component 2 values.

## Discussion

The results of this study clearly indicate that the citrus root nematode (*T. semipenetrans*), as a primary pathogen, plays a key role in both the onset and progression of tree decline. The ordinal logistic regression model showed that increased densities of second-stage juveniles and adult females significantly raised the probability of a gradual decline. These findings are in agreement with global studies reporting a direct link between nematode density and reduced tree health^32^. Notably, our results revealed that J2 density has a stronger association with citrus decline severity than adult female density, with odds ratios of 3.45 and 2.89, respectively. This indicates that J2 density serves as a stronger predictor of progression to gradual decline, highlighting the importance of monitoring early-stage nematode populations in orchard management programs. Therefore, understanding the adaptive ability of nematodes is crucial for developing suitable control strategies^33^. Notably, this study highlights the moderating role of soil physicochemical properties in this relationship.

Modeling results indicated that soil texture is a significant moderating factor influencing the impact of nematodes on tree decline. Specifically, lighter soils with higher sand content amplified the destructive effects of nematodes, likely due to physical soil characteristics that facilitate nematode movement and activity. Conversely, heavier soils with higher clay content, although potentially prone to drainage issues, appeared to exert a protective effect against nematode damage. These findings underscore the necessity of integrated management approaches that simultaneously target nematode control and soil physical improvement^34^.

In addition to soil texture, soil salinity and saturation percentage showed significant nonlinear relationships with nematode density. The generalized additive model (GAM) indicated that nematode populations peak within a specific salinity range, whereas deviations toward higher or lower salinity levels reduce their density. Similarly, increasing soil saturation beyond a certain threshold exerts an inhibitory effect on nematode populations. These findings suggest that managing soil salinity and water retention could serve as effective indirect strategies for regulating nematode densities^35^.

Another important finding of this study is the synergistic interaction between alkaline pH, high TNV, and nematode density in inducing micronutrient deficiencies, particularly zinc. In soils with high pH and substantial lime, nutrient uptake by trees is impaired, and nematode-induced root damage further intensifies this deficiency. This explains why some orchards exhibit severe decline even at moderate nematode densities. Accordingly, adjusting soil pH and implementing targeted micronutrient fertilization in calcareous soils could enhance tree resilience against nematode infestation^36^.

Analysis of latent soil components further revealed that two principal dimensions—physical and chemical—have a decisive influence on infection status and decline severity. This finding underscores the importance of adopting a systemic perspective in managing citrus orchard soils. Focusing solely on a single factor, such as nematode density or a specific soil property, is insufficient for developing comprehensive control strategies; instead, the complex interactions among multiple dimensions must be considered.

Overall, the modeling conducted in this study indicates that citrus decline cannot be attributed to a single cause. Rather, it arises from intricate interactions between biotic factors, such as nematodes, and abiotic factors, including soil physicochemical properties. Understanding these interactions provides a scientific basis for integrated management strategies. Such strategies should encompass nematode population control through biological or chemical methods, soil fertility enhancement, salinity and pH management, soil texture improvement where feasible, and the use of tolerant rootstocks and cultivars^37^. Implementing these measures in an integrated manner can reduce losses due to decline, ensure sustainable production in citrus orchards of the studied provinces and similar regions, and minimize reliance on single-factor, high-risk approaches such as excessive chemical pesticide application. This study represents a step toward a more comprehensive understanding of complex plant disease dynamics and the development of sustainable agricultural management frameworks.

## Conclusion

Sustainable management of citrus decline requires an integrated approach. Key strategies include the control of nematode populations through biological methods, the use of tolerant cultivars, and the improvement of soil physicochemical properties. Practical interventions may involve amending light-textured soils, reducing soil salinity to optimal levels, and correcting alkaline pH using amendments such as elemental sulfur. Moreover, ensuring adequate micronutrient availability, particularly in calcareous soils, can prevent deficiency symptoms. Implementing these integrated measures can both mitigate losses and enhance overall orchard productivity.

## Data Availability

All data generated or analysed during this study are included in this article.

## Abbreviations

AUC: Area under the curve
EC: Electrical conductivity
EDF: Effective degrees of freedom
FAO: Food and Agriculture Organization
GAM: Generalized additive model
GCV: Generalized cross-validation
ILR: Isometric log-ratio
J2: Second-stage juveniles
LDA: Linear discriminant analysis
MANOVA: Multivariate analysis of variance
OLR: Ordinal logistic regression
OM: Organic matter
PLS-DA: Partial least squares discriminant analysis
ROC: Receiver operating characteristic
TNV: Total neutralizing value
